# Analysis of Basketball Referee Decision-Making Using the DMQ-II Questionnaire

**DOI:** 10.3390/sports13080270

**Published:** 2025-08-15

**Authors:** Raúl Nieto-Acevedo, Carlos García-Sánchez, Moisés Marquina Nieto, Daniel Mon-Lopez, Andrea Hortiguela-Herradas, Jorge Lorenzo-Calvo

**Affiliations:** 1 Faculty of Sports Sciences, Universidad Alfonso X el Sabio, 28691 Villanueva de la Cañada, Spain; 2 Deporte y Entrenamiento Research Group, Facultad de Ciencias de la Actividad Física y del Deporte, Universidad Politécnica de Madrid, 28040 Madrid, Spain; c.gsanchez@upm.es (C.G.-S.); moisesmn95@gmail.com (M.M.N.); daniel.mon@upm.es (D.M.-L.); andrea.hortiguela.herradas@alumnos.upm.es (A.H.-H.); jorge.lorenzo@upm.es (J.L.-C.)

**Keywords:** psychological stress, officials’ health, sports integrity, mental training, decision-making fatigue

## Abstract

Basketball is a sport whose regulations require quick and complex refereeing decisions. Since the inception of sports, research on sports judgment and refereeing has been consistently present in the scientific literature. However, decision-making and the psycho-emotional factors that influence it remain somewhat unexplored in studies. The decision-making of the basketball referee has been analyzed using the DMQ II questionnaire. To achieve this, 58 referees from the Spanish Basketball Federation collaborated, comprising 45 men and 13 women, all between the ages of 18 and 38 (M = 26.5, SD = 5.5). Participants completed the 31-question DMQ II questionnaire online, yielding results with significant differences based on age and gender about stress in decision-making. A modification of the referee’s teaching methodology that includes psychological strategies is proposed both in the initiation course and throughout the refereeing career. The findings highlight the need for mental health support systems for referees, particularly addressing stress management in young and female officials. This aligns with global efforts to safeguard psychological well-being in sports professionals.

## 1. Introduction

Basketball is a complex, fast-paced sport that requires one to three referees to ensure rules are followed. Since the origins of the sport, there has been interest in training and evaluating referees—the authoritative figures on the court [1]. Referees play a vital role not only in maintaining the flow and safety of the game but also in influencing player behavior and potentially the outcome of a match [2]. In such a dynamic environment, referees must possess strong rule knowledge, physical fitness, and effective positioning [3]. Studies also highlight additional key traits: concentration, decision-making speed, anticipation, impartiality, and collaboration with fellow officials [4]. Among these, decision-making stands out as a core skill, given the many situations that must be quickly assessed during a game [5]. Physical performance directly impacts this decision-making process. Internal and external load variables are used to measure the physical effort required of referees, and evidence suggests that as workload increases, decision-making accuracy may decline [6]. Moderate physical demands, in particular, are associated with a higher chance of mistakes [7]. Beyond physical and cognitive demands, psycho-emotional factors also affect the quality of refereeing performance. Stress, anxiety, and loss of confidence caused by perceived or actual mistakes can impair performance and even lead to professional dropout [8]. Guillén and Feltz (2011) [4] proposed a conceptual model of referee self-efficacy, which includes dimensions such as rule knowledge, decision-making, communication, game control, and physical condition. Despite their importance, psycho-emotional variables remain underexplored in sports psychology research [9]. Strong self-efficacy beliefs are linked to positive emotions, which can help improve performance under pressure [10]. However, referees face many emotional stressors: media scrutiny, crowd pressure, attempts at deception by players, mistreatment by players and coaches, and gender-based discrimination [11]. These experiences can vary based on age, experience, and gender, adding complexity to each referee’s psychological profile [12]. There are tools to analyze referee movements during a game based on mechanics, infractions, fouls, teamwork, and game control. A notable example is the IOVAB instrument [13]. Using this tool requires experts—either referees’ technicians viewing the match live or from recordings—to provide detailed assessments. This is complemented by referees’ self-assessment at the end of the game to compare their perception of performance and the technical evaluation provided by officiating staff [7]. However, such tools do not address referees’ psycho-emotional needs for sound decision-making. While tools like the IOVAB evaluate observable actions and collaboration, they do not capture psychological or emotional factors. For this, instruments like the Decision-Making Questionnaire II (DMQ-II), adapted from Mann et al. (1997) [14], analyze psychological aspects such as decision-making stress, speed under uncertainty, and commitment [15]. Currently, referee training mainly emphasizes mechanics, signaling, and rule knowledge. However, these alone are insufficient. Referees need tools to manage stress and make effective decisions, even in challenging conditions. Furthermore, referee professional development should extend beyond certification through ongoing assessment and support [16]. Therefore, this study aims to analyze the psychological dimensions of referees using the DMQ-II, focusing on three key variables: gender, age, and experience in senior-level competitions. By exploring these dimensions, we hope to better understand how individual and contextual factors influence referees’ psychological profiles and decision-making in basketball.

## 2. Materials and Methods

### 2.1. Participants

The sample includes 58 referees from the 1st National category of the Spanish Basketball Federation, consisting of 45 men and 13 women (see Table 1). A total of 103 referees who met the criteria for officiating in the Spanish Basketball Federation (FEB) were contacted. Out of these, 58 responded and completed the questionnaire. All participants were informed about the study’s objectives, the voluntary nature of their participation, and the confidentiality and anonymity of the collected data. Before participating, each referee signed an informed consent form following the ethical standards outlined in the Declaration of Helsinki. The study was reviewed and approved by the Universidad Politécnica de Madrid Ethics Committee for Research Involving Human Subjects (FDRED00000-DML-DATOS-20230609), ensuring that all procedures adhered to ethical principles for research in social and behavioral sciences.

### 2.2. Procedures

The referees completed the validated Decision Making Questionnaire (DMQ II. Exhibit 1) [17] online, translated into Spanish by Barbero et al. [14]. Those responsible for the federation’s referee training group were informed, and approved the administration of the questionnaire. It was completed using Google Forms and sent only once. There was a 78.4% participation rate in the study.

This is a 31-item questionnaire that defines six scales, which assess six distinct behavioral patterns, defined by the author as: vigilance, hypervigilance/panic, defensive avoidance, rationalization, transference, and delay. For this instrument to more accurately assess decision-making style in the context of sports refereeing, the wording of the items was appropriately modified to adapt it to the specific situations that a basketball referee must face.

These scales correspond to the three factors that make up the questionnaire:

Factor 1. Stress in decision-making
Factor 2. Rapid decision-making under uncertainty
Factor 3. Determination and commitment in decision-making

The questionnaire consists of 31 items, each evaluated using a Likert scale. For scoring purposes, each response was assigned a numerical value: 4 for “always,” 3 for “almost always,” 2 for “sometimes,” 1 for “rarely,” and 0 for “never.”

A descriptive principal components analysis was conducted, given that the questionnaire is structured around these three factors:

Factor 1: Stress in decision-making—This factor includes the following items: 1, 2, 5, 6, 8, 9, 10, 12, 16, 17, 21, 23, 24, 26, 27, 29, 30, and 31.

Factor 2: Rapid decision-making under uncertainty—This factor includes items: −3, 4, −7, −14, 18, and 20. The negative sign (“−”) indicates that the item has an inverse relationship with the factor, contrary to what might be inferred from its wording.

Factor 3: Determination and commitment in decision-making—This factor is defined by items 11, 15, 19, 22, and 28 [11].

### 2.3. Statistical Analysis

The three factors were analyzed by gender (men and women), age (18–24 years, 25–30 years and >30 years), and experience refereeing senior categories (0–3 years, 4–8 years and >8 years). All data are presented as the mean ± SD. Normal distribution of the data was confirmed using the Shapiro–Wilk test, except for factor 2 (quick decision with uncertainty) and factor 3 (determination and commitment in decision-making). The independent sample *t*-test was applied to parametric variables and the Mann–Whitney test to nonparametric variables to determine whether there were any significant differences in the performance of the three factors by gender. Levene’s test was used to verify the homogeneity of the variables analyzed. A one-way analysis of variance (ANOVA) or Kruskal–Wallis nonparametric test was used to compare between-group differences (i.e., groups of age and years of experience), followed by a post hoc correction which was implemented when it was significant. Hedges g ES were used in *t*-test to calculate and interpreted following previous guidelines: trivial (0.20), small (0.20–0.59), moderate (0.60–1.19), large (1.20–2.00), very large (2.00–4.00), and extremely large (4.00). To quantify the magnitude of the observed effects, eta squared (η^2^) was calculated as a measure of effect size. Values of η^2^ were interpreted according to the conventional benchmarks: small (η^2^ = 0–0.05), medium (η^2^ ≥ 0.06), and large (η^2^ ≥ 0.14), as proposed by Cohen (1988) [18]. To check the reliability of the questionnaire, Cronbach’s alpha coefficient has been used, obtaining a value of 0.783, which indicates that the reliability of the questionnaire is good. Statistical significance was set a priori to *p* < 0.05. All reliability assessments were performed using Jeffreys’s Amazing Statistics Program (JASP) open-source software (version 0.12.2.0, University of Amsterdam, Amsterdam, The Netherlands).

## 3. Results

**Figure 1:**
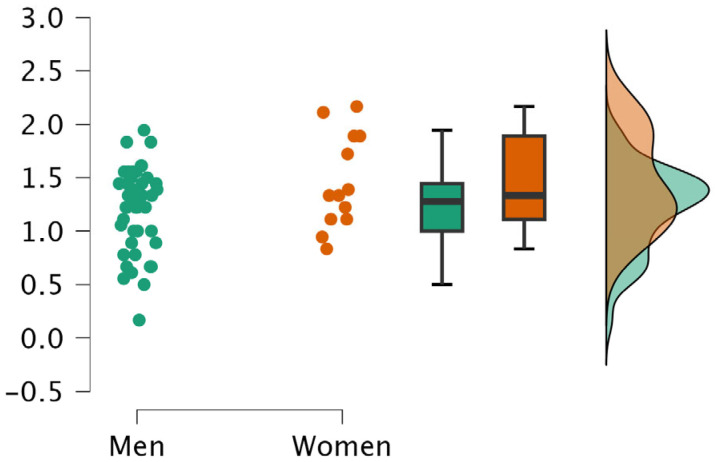
Gender differences in stress in decision-making.

An independent *t*-test revealed significantly higher stress levels (Factor 1) in women compared to men (t = −2.04, *p* = 0.046, ES = −0.64; see Figure 1). However, no significant differences were found in Factor 2—Quick Decision (positive and negative) (*p* = 0.282 and 0.955, respectively) or in Factor 3—Determination (*p* = 0.133).

**Figure 2:**
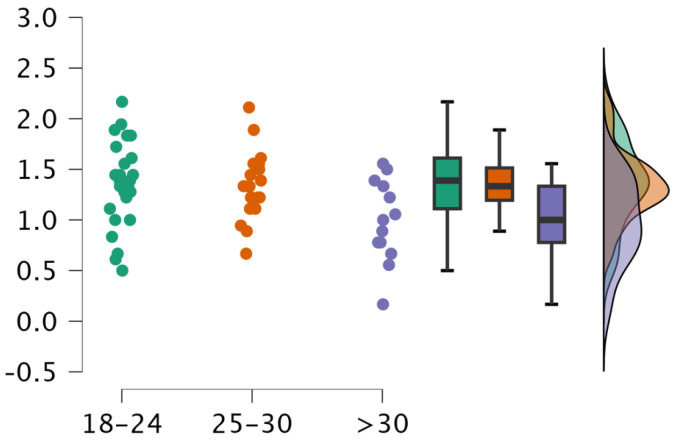
Age group differences in stress in decision-making.

Significant differences were observed between groups of age in factor 1 (stress). Groups of 18–24 years and 25–30 years demonstrated significantly higher values than >30 years (*p* = 0.025 and 0.046, respectively, see Figure 2). In contrast, no significant differences were found in the other two factors (*p* > 0.05, see Table 2).

The ANOVA test revealed no significant differences among the three groups based on years of experience in senior categories across any of the three factors (Table 2).

## 4. Discussion

This study revealed significant differences in decision-making factors based on gender and age, while refereeing experience did not have a significant effect on any of the three factors analyzed. Female and younger referees both reported higher stress levels, though these patterns must be interpreted within broader psychological and social contexts.

Regarding gender, female referees reported significantly higher stress levels than their male counterparts (t = −2.04, *p* = 0.046, ES = −0.64), which aligns with previous research suggesting that women may experience greater psychological strain in high-pressure officiating contexts [11]. However, it is important to consider that this elevated stress may not be solely internal or dispositional. The sports environment itself—often male- dominated—can be more hostile, scrutinizing, or less supportive toward female referees, potentially contributing to heightened stress perceptions. Some studies report no gender- related differences in stress or decision-making performance [19,20], indicating that factors such as competition level, organizational support, and role clarity could serve as moderators.

It is also important to note that higher stress levels do not necessarily lead to decreased performance. In some cases, increased arousal can improve focus and decision accuracy. Therefore, implying that female referees perform worse under stress risks reinforcing gender stereotypes and should be avoided. Instead, it underscores the need for more inclusive environments and targeted support.

In terms of age, younger referees (18–30 years) reported significantly higher stress levels than those over 30. This may be due to less experience with complex game situations, lower confidence in handling pressure, and a limited ability to regulate emotions under demanding conditions [21]. Additionally, older referees may benefit from a perceived authority or leadership role on the court, which contributes to composure and confidence during decision-making [22].

Contrary to expectations, refereeing experience—measured in years officiating in senior categories—was not associated with significant differences in any of the decision-making dimensions. This result contrasts with the literature that emphasizes the positive effects of accumulated experience on emotional regulation and decision-making quality [8,23]. One possible explanation is that “experience,” defined only by years, may not capture its qualitative dimension—such as the level of competition, frequency of officiating, or types of pressure faced. Furthermore, the role of self-efficacy in referee performance—though not directly measured in this study—has been widely documented. Research has shown that self-efficacy mediates the relationship between stress, experience, and decision-making success [8,10].

Several studies highlight the term self-efficacy as a necessary variable for good refereeing performance [8,10]. López-Aguilar et al. [20] highlight that referees with less than 4 years of experience have lower levels of self-efficacy than those with more experience. Guillén and Feltz [8] began their research with a conceptual model of the self-efficacious referee, which they called referee efficacy and defined as the extent to which referees believe they have the capacity to perform their job successfully. Similarly, MacMahon et al. [24] highlighted that decision-making is influenced by memories of past performances—a variable directly tied to cumulative experience.

Interestingly, while age and experience often overlap, this study observed that age—but not experience—was linked to stress differences. Some research supports the idea that younger referees are considered to be more stressed regarding the possibility of making mistakes [21]. In addition, the presence of a referee as a leader (which would facilitate good performance of the game) correlated with age. This HE has to ensure that a referee is in their best moment when their age is elderly compared to that of the player [22]. Therefore, older referees, even if they do not have the most experience, will have fewer difficulties in ensuring the smooth running of the match. Nevertheless, other studies, such as López-Aguilar et al. [20], found no age-related differences in self-efficacy, suggesting that while age may support stress regulation, the development of self-efficacy is more closely linked to the nature and quality of the experience rather than age itself.

In light of these findings, referee training programs should not only focus on rules and mechanics but also incorporate the development of psychological skills. Moreover, structural changes to improve the inclusivity and safety of the refereeing environment—particularly for women—could help mitigate stress factors that are systemic rather than individual.

This study presents several limitations that should be considered when interpreting the results. Firstly, in terms of gender distribution, there is an imbalance between male (*n* = 45) and female (*n* = 13) referees. This may limit the generalizability of the findings, especially regarding gender-based comparisons. While the sample size and level of officiating are adequate for preliminary insights, future studies should aim for more balanced representation. Secondly, all participants belonged to the Spanish Basketball Federation. Cultural and structural differences in other federations or countries may lead to different psychological outcomes. Finally, the cross-sectional design of the study does not allow for causal conclusions or observation of changes over time.

### Practical Implications

The present findings suggest that referee education and development programs should integrate psychological skills training alongside technical and rules-based instruction, with particular emphasis on stress management and emotional regulation. Female referees may benefit from targeted support strategies and inclusive organizational policies to address systemic pressures present in male-dominated environments. For younger referees, mentorship programs and graduated exposure to high-pressure game situations could help build confidence and reduce stress vulnerability. Given that years of experience alone did not predict decision-making outcomes, training interventions should focus on the quality and context of officiating experience—such as competition level, frequency, and type of matches—rather than solely on tenure. These measures could contribute to enhancing decision-making performance, promoting referee well-being, and fostering more equitable and supportive officiating environments.

## 5. Conclusions

This study highlights that decision-making stress in basketball referees is significantly influenced by gender and age, but not by refereeing experience—at least not in the way experience was operationalized here. Female referees tend to experience higher stress levels, which may impact their performance under pressure. Younger referees are also more susceptible to stress, possibly due to limited exposure to high-stakes situations or lower confidence.

In contrast, refereeing experience did not significantly predict differences in stress or decision-making attributes. This suggests that experience alone, when measured in years, may not be a reliable indicator of psychological readiness or decision-making quality. Age appears to contribute more meaningfully—perhaps due to maturity, leadership perception, or emotional regulation—but experience quality and context may ultimately be more decisive than duration.

Overall, these findings underline the importance of considering age and gender-related psychological dynamics in referee training programs. Future research should adopt more refined measures of experience, incorporate variables such as self-efficacy, and explore how contextual and situational factors interact with psychological skills to shape decision-making performance on the court.

## Figures and Tables

**Table 1:** Descriptive Statistics of participants.

		M	SD
Age	Men	27.0	6.2
	Women	24.5	3.3
Total experience as a referee	Men	7.7	4.8
	Women	5.85	2.0
Total experience in senior categories	Men	5.9	5.1
	Women	4.2	2.1

Note. M= Mean; SD = Standard Deviation.

**Table 2:** Results of ANOVA and Kruskal–Wallis Tests with Post Hoc Comparisons for Decision-Making Factors by Age and Senior-Level Refereeing Experience.

Age	Factor 1. Stress in Decision-Making					Factor 2. Positive Quick Decision with Uncertainty					Factor 2. Negative Quick Decision with Uncertainty					Factor 3. Determination and Commitment in Decision Making				
	M	SD	Post Hoc Comparison	*p*-Value	ES	M	SD	Post Hoc Comparison	*p*-Value	ES	M	SD	Post Hoc Comparison	*p*-Value	ES	M	SD	Post Hoc Comparison	*p*-Value	ES
18–24 (*n* = 25)	135	0.43	vs. 25–30	0.98	0.05	1.00	0.42	vs. 25–30	0.26	−0.23	2.97	0.51	vs. 25–30	1.00	−0.02	2.19	0.30	vs. 25–30	1.00	0.01
25–30 (*n* = 20)	133	0.33	vs. >30	**0.03**	0.92	1.25	0.56	vs. >30	0.52	0.15	2.93	0.71	vs. >30	0.33	−0.21	2.18	0.29	vs. >30	0.27	−0.18
>30 (*n* = 13)	0.99	0.41	vs. 18–24	**0.05**	0.87	1.03	0.54	vs. 18–24	0.74	−0.04	3.31	0.46	vs. 18–24	0.22	−0.24	2.29	0.34	vs. 18–24	0.37	−0.18
Year of experience in senior categories																				
0–3 (*n* = 26)	1.28	0.41	vs. 4–8	0.82	−0.18	1.18	0.46	vs. 4–8	0.68	0.16	2.86	0.63	vs. 4–8	0.24	−0.23	2.17	0.32	vs. 3–8	1.00	−0.10
4–8 (*n* = 21)	1.35	0.44	vs. >8	0.36	0.50	0.98	0.54	vs. >8	1.00	0.16	3.11	0.52	vs. >8	1.00	−0.09	2.25	0.26	vs. >8	1.00	−0.03
>8 (*n* = 11)	1.08	0.33	vs. 0–3	0.18	0.67	1.09	0.52	vs. 0–3	1.00	0.04	3.30	0.51	vs. 0–3	0.10	−0.28	2.24	0.37	vs. 0–3	1.00	−0.11

Note: In bold, significant differences *p* < 0.05.

## Data Availability

The datasets generated and analyzed during the current study are not publicly available due to privacy and ethical considerations.

## References

[1] Bustos J.D. (2021). Competency-Based Curriculum Design for the Training of Basketball Referees. Master’s Thesis.

[2] Philippe F.L., Vallerand R.J., Andrianarisoa J., Brunel P. (2009). Passion in Referees: Examining Their Affective and Cognitive Experiences in Sport Situations. J. Sport Exerc. Psychol..

[3] Aginsky K.D. (2010). Why It Is Difficult to Detect an Illegally Bowled Cricket Delivery with Either the Naked Eye or Usual Two-Dimensional Video Analysis. Br. J. Sports Med..

[4] Guillén F., Jiménez H. (2001). Characteristics Desirable in Arbitration and the Judgment Sports. Mag. Psychol. Sport.

[5] Diotaiuti P., Falese L., Mancone S., Purromuto F. (2017). A Structural Model of Self-Efficacy in Handball Referees. Front. Psychol..

[6] García-Santos D., Pino-Ortega J., García-Rubio J., Cowgirl T.O., Ibáñez S.J. (2019). Internal and External Demands in Basketball Referees during the U-16 European Women’s Championship. Int. J. Environ. Res. Public. Health.

[7] García-Santos D., Gómez-Ruano M.A., Cowgirl A., Ibáñez S.J. (2020). Systematic Review of Basketball Referees’ Performances. Int. J. Perform. Anal. Sport.

[8] Guillén F., Feltz D. (2011). A Conceptual Model of Referee Efficacy. Front. Psychol..

[9] Jaenes C.C., Bohórquez M.R., Caracuel J.C., López A.M. (2012). Emotional State and Stress Situations in Basketball Referees. J. Sport Psychol..

[10] Johansen B.T., Ommundsen Y., Haugen T. (2018). Referee Efficacy in the Context of Norwegian Soccer Referees—A Meaningful Construct. Psychol. Sport Exerc..

[11] Aguirre-Loaiza H., Holguín J., Arenas J., Núñez C., Barbosa-Granados S., García-Mas A. (2020). Psychological Characteristics of Sports Performance: Analysis of Professional and Semi-Professional Football Referees. J. Phys. Educ. Sport.

[12] Sánchez J., Serrat S., Castillo E., Nuviala A. (2021). Confirmatory Factor Analysis and Validity of the Sexual Harassment Scale in Football Refereeing. Int. J. Environ. Res. Public. Health.

[13] García-Santos D., Ibáñez S.J. (2016). Design and Validation of a Instrument of Observation for the Evaluation of a Basketball Referee (IOVAB). SPORT TK-Euro-Am. J. Sports Sci..

[14] Barbero Y., Vila E., Maciá A., Pérez-Llantada C., Navas M.J. (1993). Spanish Adaptation of Leon Mann’s DMQ-II Questionnaire. J. Gen. Appl. Psychol..

[15] Gimeno F., Buceta J.M., Lahoz D., Sanz G. (1998). Evaluation of the Decision-Making Process in the Context of Sports Refereeing: Psychometric Properties of the Spanish Adaptation of the DMQ II Questionnaire in Handball Referees. J. Sport Psychol..

[16] Sabag E., Lidor R., Arnon M., Morgulev E., Bar-Eli M. (2023). Teamwork and Decision Making among Basketball Referees: The 3PO Principle, Refereeing Level, and Experience. J. Hum. Kinet..

[17] Mann L., Burnett P., Radford M., Ford S. (1997). The Melbourne Decision Making Questionnaire: An Instrument for Measuring Patterns for Coping with Decisional Conflict. J. Behav. Decis. Mak..

[18] Cohen J. (2013). Statistical Power Analysis for the Behavioral Sciences. Statistical Power Analysis for the Behavioral Sciences.

[19] García-Santos D., Gamonales J.M., León K., Muñoz J. (2017). A Case Study: Characterization of Physiological, Kinematic and Neuromuscular Demands of Handball Referee during Competition. E-Handball Mag. Sci. Sport.

[20] Lopez-Aguilar J., Castillo-Rodriguez T.O., Chinchilla-Minguet J.L., Onetti-Onetti W. (2021). Relationship between Age, Category and Experience with the Soccer Referee’s Self-Efficacy. PeerJ.

[21] Kaissidis-Rodafinos A., Anshel M.H. (1993). Sources of and Responses to Acute Stress in Adults and Adolescent Australian Basketball Referees. Aust. J. Sci. Med. Sport.

[22] Castagna C., Abt G., D’Ottavio S., Weston M. (2005). Age-Related Effects on Fitness Performance in Elite-Level Soccer Referees. J. Strength Cond. Res..

[23] Nurcahya Y., Kusumah W., Mulyana D., Kodrat H. (2021). Analysis of Football Referee Satisfaction in Making Decision Based on Experience Levels. J. Pendidik. Jasm. Dan Olahraga.

[24] MacMahon C., Starkes J., Deakin J. (2007). Referee Decision Making in a Video-Based Infringement Detection Task: Application and Training Considerations. Int. J. Sports Sci. Coach..

